# Mathematical Modelling with the Exact Solution of Three Different Bioheat Conduction Models of a Skin Tissue Shocked by Thermoelectrical Effect

**DOI:** 10.1155/2023/3863773

**Published:** 2023-07-17

**Authors:** Eman A. N. Al-Lehaibi

**Affiliations:** Mathematics Department, Al-Lith University College, Umm Al-Qura University, Al-Lith, Saudi Arabia

## Abstract

This research deals with the temperature increment and responsiveness of skin tissue to a continuous flow of surface heat induced by a constant-voltage electrical current. The exact analytical solution for the dual-phase-lag (DPL) of bioheat transfer has been obtained. It is used to confine the variables to a limited domain to solve the governing equations. The transition temperature reactions have been measured and investigated. The figures provide a comparison of the Pennes, Tzou models, and Vernotte–Cattaneo models. The numerical results demonstrate the values of the voltage, resistance, electric shock time, and dual-phase-lag time parameters which have significant influences on the distributions of the dynamic and conductive temperature rise through the skin tissue.

## 1. Introduction

Many methods of treatment and application rely substantially on temperature transfer through living skin tissue [1]. Lasers, microwaves, and other technological breakthroughs have also contributed to the progress of biothermal transfer. Several experts, including Pennes's works, weigh in on bioheat transmission research in living biological tissues [2]. He refined the parabolic equation of the model for living tissues and introduced the first bioheat transfer model. Pennes's biological transmission technique has also been used to describe the consistency and nature of temperature action in live bodies and biological tissues. On the exterior, such irregular results demonstrate a hyperbolic conduction behaviour which is called the non-Fourier model of heat conduction. Vernotte and Cattaneo's (V-C) multiple modulations of the heat conduction law were given as a linear thermal extension form of the well-known Fourier law to describe this kind of hyperbolic differential equation. To analyze the influence of microwave and flux thermal activity, they created a thermal wave model [3, 4]. Many methods were tried to cure different types of skin tissue problems without hurting the good tissue around or near them. Xu et al. obtained the solution of Pennes's bioheat transfer equation (PBTE) analytically. In addition, they investigated skin bioheat transfer, skin biomechanics, thermal injury, and skin structure [5]. The dual-phase-lag, hyperbolic, and parabolic biomass transport models were used to investigate the thermomechanical activity based on the non-Fourier skin tissues under different surface thermal loading constraints. Xu and colleagues found substantial discrepancies between Pennes's thermal wave and dual-phase-lag (DPL) anticipation models [6]. Furthermore, Rossmanna and Haemmerich investigated how the thermal characteristics, perfusion, and dielectric properties of biological tissues vary with temperature at hyperthermic and ablation temperatures [7]. Moradi et al. have focused on the temperature change in skin tissue caused by heating with a time-dependent surface [8]. The model of Tzou was expanded by using the dual-phase-lag (DPL) technique, which considers the delayed activity in a high rate of reaction. Despite the lengthy operation, the small-scale response was discovered in good time [9, 10]. O¨zis¸ik and Tzou have introduced phase lag behaviour for a temperature gradient [11]. Askarizadeh and Ahmadikia used the dual-phase-lag (DPL) model for solving transient heat transmission difficulties in skin tissue [12].

Non-Fourier thermal activity was used in the Liu and Chen heat conduction of the dual-phase-lag (DPL) model to assess the thermal transit in biological tissue and to diagnose hyperthermia [13]. Zhang defined phase lag or relaxation periods in terms of tissue and blood properties, the interphase charge of the parameter of heat transfer, and perfusion rate [14]. He discovered that the lap durations for living tissues are identical. Dutta and Kundu conducted a study on thermal wave propagation for a changing and constant thermal flow through a skin tissue surface to detect hyperthermia [15].

To analyze the increment distribution of deep skin tissues temperature in a human leg, Agrawal and Pardasani suggested utilizing a finite element approach [16]. Kumar and Rai investigated the thermal behaviour using the dual-phase-lag (DPL) model based on time-fractional consideration [17]. Moradi et al. used the dual-phase-lag (DPL) bioheat transfer model with an analytical approach on skin tissue as a finite domain of cosine, continuous, and pulse thermal flow conditions on the skin's bounding surface [8].

Liu and Xu improved a different analytical approach for Pennes's bioheat equation solution for fluctuations in the temperature of skin tissue due to the sinusoidal thermal flow [18]. Shih et al. overcame the impacts of studying the temperature reaction on the biological skin tissue under sine heat flux [19]. The exact analytical solution of Pennes's bioheat and hyperbolized bioheat transfer models for periodic constant and pulsed train heat flux boundary conditions has been provided by Ahmadikia et al. [20]. To characterize the impacts of the pulsatile blood flow in thermal disruption, Horng et al. used a statistical model [21]. He observed that the thermal region of the tumor had a pure little variation on the continuum range of blood from usual to parabolic blood flow velocity profiles. Shih et al. investigated how the thermal relaxation time interacts with the pulsatile blood flow during thermal treatments in living tissues [22]. Youssef and Alghamdi obtained the solution to one-dimensional problems of thermoelastic dual-phase-lag (DPL) skin tissue based on specific temperature stress [23]. Kundu and Dewanjee pioneered the response of a non-Fourier thermal approach in one layer of skin tissue [24]. Ezzat and Alabdulhadi introduced a novel mathematical model of a generalized thermoviscoelasticity theory based on Pennes's bioheat transfer equation with a dual phase-lag to investigate the biothermomechanics behaviour in the skin tissue [25]. Ezzat constructed the model of the thermoviscoelasticity theory of fractional dual-phase-lag heat conduction law with rheological properties of the volume to investigate one-dimensional bioheat transfer and heat-induced mechanical response in human skin tissue [26]. In Pennes's bioheat transmission and heat-induced mechanical response in human living tissue with variable thermal conductivity based on rheological parameters of the volume, Ezzat presented a novel notion of memory-dependent derivative [27]. Many applications of analytical analysis of the dual-phase-lag model of bioheat transfer have been constructed and solved [28–32].

In the current work, the exact solutions of a novel mathematical model of skin tissue will be obtained in the framework of the dual-phase-lag (DPL) heat conduction model when the bounding plane of the surface of the skin tissue is exposed to a continuous heat flux due to a constant voltage of the thermoelectric effect. To obtain the exact analytical solutions, variables will be separated into a finite domain. The influence of the dual-phase-lag time parameters, voltage value, resistance value, and electric shock time value will be investigated and discussed. Comparing the three different models of bioheat conduction is the main goal of this work and is a novel effort.

## 2. Basic Equations

The model of bioheat transmission was created to investigate the time-dependent temperature increment caused by a heat source or thermal loading. Pennes created the first biological tissue model in the context of the classical Fourier's law of heat conduction as follows [23, 33–36]:

(1) $$\begin{array}{c} \nabla^{2}T=\frac{\rho C}{K}\frac{\partial T}{\partial t}+\frac{\rho_{b}w_{b}C_{b}}{K}\left(T-T_{b}\right)-\frac{1}{K}\left(Q_{met}+Q_{ext}\right)\text{,} \end{array}$$

where *ρ*_*b*_, *w*_*b*_, *C*_*b*_, and *T*_*b*_ are the blood density, blood perfusion rate, specific heat of the blood, and blood temperature, respectively. *K*, *ρ*, and *C* are the thermal conductivity, density, and specific heat of the skin tissue. *T* denotes the absolute temperature function. *Q*_met_ is the metabolic heat generated by the chemical reaction inside the skin tissue, and it is assumed to be a constant, while *Q*_ext_ is the external heat source and it could be a function. Finally, ∇^2^ is the well-known Laplace operator.

Vernotte and Cattaneo (V-C) have updated the classic Fourier law of thermal transfer by positioning the assumption of the finite and limited speed of the propagation of the thermal wave and utilizing the following hyperbolic heat conduction form [23, 33–36]:

(2) $$\begin{array}{c} \nabla^{2}T=\frac{\rho C}{K}\left(\tau_{q}\frac{\partial }{\partial t}+1\right)\frac{\partial T}{\partial t}+\frac{\rho_{b}w_{b}C_{b}}{K}\left(\tau_{q}\frac{\partial }{\partial t}+1\right)\left(T-T_{b}\right)-\frac{1}{K}\left(\tau_{q}\frac{\partial }{\partial t}+1\right)\left(Q_{met}+Q_{ext}\right)\text{,} \end{array}$$

where *τ*_*q*_=(*α*/*c*_0_^2^) > 0 is defined as the relaxation time parameter, *c*_0_ gives the thermal wave's speed inside the medium, and *α* gives the thermal diffusivity.

The dual-phase-lag (DPL) heat conduction equation is based on the dual response between the temperature gradient and heat flow, which modifies the heat conduction equation of Tzou's classical Fourier's.

Then, in this case, the heat conduction equation takes the following form [23, 33–36]:

(3) $$\begin{array}{c} \left(\tau_{T}\frac{\partial }{\partial t}+1\right)\nabla^{2}T=\frac{\rho C}{K}\left(\tau_{q}\frac{\partial }{\partial t}+1\right)\frac{\partial T}{\partial t}+\frac{\rho_{b}w_{b}C_{b}}{K}\left(\tau_{q}\frac{\partial }{\partial t}+1\right)\left(T-T_{b}\right)-\frac{1}{K}\left(\tau_{q}\frac{\partial }{\partial t}+1\right)\left(Q_{met}+Q_{ext}\right)\text{,} \end{array}$$

where *τ*_*T*_ ≥ 0 is the second relaxation time parameter, which gives the phase lag time of the temperature gradient.

We assume the temperature increment's function to take the following form [35]:

(4) $$\begin{array}{c} \theta =T-T_{b}\text{.} \end{array}$$

Then, we obtain the heat conduction form as follows:

(5) $$\begin{array}{c} \left(\tau_{T}\frac{\partial }{\partial t}+1\right)\nabla^{2}\theta =\frac{\rho C}{K}\left(\tau_{q}\frac{\partial }{\partial t}+1\right)\frac{\partial \theta }{\partial t}+\frac{\rho_{b}w_{b}C_{b}}{K}\left(\tau_{q}\frac{\partial }{\partial t}+1\right)\theta -\frac{1}{K}\left(\tau_{q}\frac{\partial }{\partial t}+1\right)\left(Q_{met}+Q_{ext}\right)\text{.} \end{array}$$

## 3. Problem Formulation

**Figure 1:**
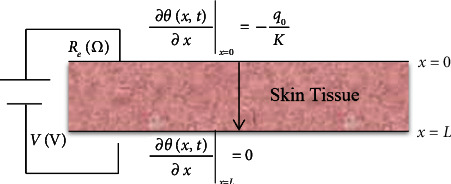
The skin tissue is subjected to a thermoelectrical loading.

We assume that the region of a skin tissue 0 ≤ *x* ≤ *L* obeys the dual-phase-lag formulation as in equation (5) (see Figure 1).

The medium is initially quiescent and has no external heat source i.e., *Q*_ext_=0, while *Q*_met_ is a constant.

We consider the surface of the skin tissue *x*=0 to be subjected to constant heat flux *q*_0_, while the surface of the other side *x*=*L* has a zero-heat flux.

Thus, the heat conduction model takes the following form [35]:

(6) $$\begin{array}{c} \left(\tau_{T}\frac{\partial }{\partial t}+1\right)\frac{\partial^{2}\theta }{\partial x^{2}}=\left(\frac{\tau_{q}\rho C}{K}\frac{\partial^{2}}{\partial t^{2}}+\frac{\left(\rho C+\tau_{q}\;\rho_{b}w_{b}C_{b}\right)}{K}\frac{\partial }{\partial t}+\frac{\rho_{b}w_{b}C_{b}}{K}\right)\theta -\frac{Q_{met}}{K}\text{.} \end{array}$$

The initial conditions take the following form:

(7) $$\begin{array}{c} {\left.\theta \left(x,t\right)\right|}_{t=0}={\left.\frac{\partial \theta \left(x,t\right)}{\partial t}\right|}_{t=0}=0\text{.} \end{array}$$

The boundary conditions take the following form:

(8) $$\begin{array}{c} {\left.\frac{\partial \theta \left(x,t\right)}{\partial x}\right|}_{x=0}=-\frac{q_{0}}{K}\;{\left.\text{and }\frac{\partial \theta \left(x,t\right)}{\partial x}\right|}_{x=L}=0\text{.} \end{array}$$

The boundary value problem (B. V. P.) in equations (6)–(8) contains nonhomogeneous partial differential equations with nonhomogeneous boundary conditions on the surface of the skin tissue. Hence, the differential equations must be formulated in two parts, i.e., a steady part and a transient part as follows [35, 37, 38]:

(9) $$\begin{array}{c} \theta \left(x,t\right)=\theta_{1}\left(x,t\right)+\theta_{2}\left(x\right)\text{.} \end{array}$$

Then, the transient part takes the following form:

(10) $$\begin{array}{c} \left[\left(\tau_{T}\frac{\partial }{\partial t}+1\right)\frac{\partial^{2}}{\partial x^{2}}-\left(\frac{\tau_{q}\rho C}{K}\frac{\partial^{2}}{\partial t^{2}}+\frac{\left(\rho C+\tau_{q}w_{b}C_{b}\rho_{b}\right)}{K}\frac{\partial }{\partial t}+\frac{w_{b}C_{b}\rho_{b}}{K}\right)\right]\theta_{1}=0\text{,} \end{array}$$

where the second part of the steady state is as follows:

(11) $$\begin{array}{c} \left(\frac{d^{2}}{dx^{2}}-\lambda^{2}\right)\theta_{2}\left(x\right)=-\gamma \;Q_{met}\text{,} \end{array}$$

where *λ*^2^=(*w*_*b*_*C*_*b*_*ρ*_*b*_/*k*) > 0 and *γ*=(1/*k*) > 0.

The steady-state part has the following boundary conditions [35]:

(12) $$\begin{array}{c} {\left.\frac{d\theta_{2}\left(x\right)}{d\;x}\right|}_{x=0}=-\frac{q_{0}}{K}\text{ and }{\left.\frac{d\theta_{2}\left(x\right)}{d\;x}\right|}_{x=L}=0\text{.} \end{array}$$

Then, according to the boundary conditions of equation (8), the solution of equation (6) is as follows:

(13) $$\begin{array}{c} \theta_{2}\left(x\right)=\frac{q_{0}\cosh\;\lambda \left(L-x\right)}{\lambda K\;\sinh\;\lambda L}+\frac{\gamma Q_{met}}{\lambda^{2}}\text{.} \end{array}$$

The initial conditions and the boundary conditions of the transient part of the differential equation (7) take the following form:

(14) $$\begin{array}{c} {\left.\theta_{1}\left(x,t\right)\right|}_{t=0}=-\theta_{2}\left(x\right),{\left.\frac{\partial \theta_{1}\left(x,t\right)}{\partial t}\right|}_{t=0}=0\text{,} \end{array}$$

and

(15) $$\begin{array}{c} {\left.\frac{\partial \theta_{1}\left(x,t\right)}{\partial x}\right|}_{x=0}={\left.\frac{\partial \theta_{1}\left(x,t\right)}{\partial x}\right|}_{x=L}=0\text{.} \end{array}$$

To get the solution of equation (10), we write the expansion of the function *θ*_1_(*x*, *t*) in the Fourier series expansion as follows [35, 37, 38]:

(16) $$\begin{array}{c} \theta_{1}\left(x,t\right)=\overset{\infty }{\underset{n=0}{\sum }}\vartheta_{n}\left(t\right)\cos\left(\frac{n\pi }{L}x\right)\text{,} \end{array}$$

where it must satisfy the boundary conditions in equation (15).

By substituting equation (16) into equation (10), we obtain the following equation:

(17) $$\begin{array}{c} \left[\frac{d^{2}}{dt^{2}}+A_{1n}\frac{d}{dt}+A_{2n}\right]\vartheta_{n}\left(t\right)=0\text{,}n=0,1,2,3\ldots \text{,} \end{array}$$

where *A*_1*n*_=(*ω*_*n*_^2^*ητ*_*T*_+(1+*τ*_*q*_*ε*)/*τ*_*q*_), *A*_2*n*_=(*ω*_*n*_^2^*η*+*ε*/*τ*_*q*_), *ε*=(*w*_*b*_*C*_*b*_*ρ*_*b*_/*ρC*), *η*=(*k*/*ρC*), and *ω*_*n*_=(*nπ*/*L*).

The solutions of equation (17) in general forms take the following form [35, 37, 38]:

(18) $$\begin{array}{c} \vartheta_{n}\left(t\right)=a_{n}f_{1}\left(k_{1n},t\right)+b_{n}f_{2}\left(k_{2n},t\right)n=0,1,2,\ldots \text{,} \end{array}$$

and from this, we have

(19) $$\begin{array}{c} \vartheta_{1}\left(x,t\right)=\overset{\infty }{\underset{n=0}{\sum }}\left[a_{n}f_{1}\left(k_{1n},t\right)+b_{n}f_{2}\left(k_{2n},t\right)\right]\cos\left(\omega_{n}x\right)\text{,} \end{array}$$

where *k*_1*n*_, *k*_2*n*_ are the solutions of the characteristic equation represented as

(20) $$\begin{array}{c} k^{2}+A_{1n}\;k+A_{2n}=0\text{.} \end{array}$$

The roots of the characteristic equation (20) take the following form:

(21) $$\begin{array}{c} k_{1n}=\frac{-A_{1n}+\sqrt{{∆}_{n}}}{2},k_{2n}=\frac{-A_{1n}-\sqrt{{∆}_{n}}}{2}\text{,} \end{array}$$

where ∆_*n*_=*A*_1*n*_^2^ − 4*A*_2*n*_.

To use the initial condition of equation (14), we must expand *θ*_2_(*x*) in a Fourier series expansion as follows:

(22) $$\begin{array}{c} \theta_{2}\left(x\right)=\frac{q_{0}}{2LK\lambda^{2}}+\frac{\gamma Q_{met}}{2\lambda^{2}}+\frac{q_{0}}{KL}\overset{\infty }{\underset{n=1}{\sum }}\frac{1}{\left(\lambda^{2}+\omega_{n}^{2}\right)}\cos\left(\omega_{n}x\right)\text{.} \end{array}$$

The initial condition of equation (14) lead to the following linear system of algebraic equations:

(23) $$\begin{array}{c} \left[a_{0}f_{1}\left(k_{10},0\right)+b_{0}f_{2}\left(k_{20},0\right)\right]\cos\left(\omega_{0}x\right)\\ +\overset{\infty }{\underset{n=1}{\sum }}\left[a_{n}f_{1}\left(k_{1n},0\right)+b_{n}f_{2}\left(k_{2n},0\right)\right]\cos\left(\omega_{n}x\right)=-\phi_{2}\left(x\right)\text{,} \end{array}$$

and

(24) $$\begin{array}{c} \cos\left(\omega_{0}x\right)\left[a_{0}\frac{\partial f_{1}\left(k_{10},0\right)}{\partial t}+b_{0}\frac{\partial f_{2}\left(k_{20},0\right)}{\partial t}\right]\\ +\overset{\infty }{\underset{n=1}{\sum }}\cos\left(\omega_{n}x\right)\left[a_{n}\frac{\partial f_{1}\left(k_{1n},0\right)}{\partial t}+b_{n}\frac{\partial f_{2}\left(k_{2n},0\right)}{\partial t}\right]=0\text{.} \end{array}$$

In the case of ∆_*n*_ > 0, we have

(25) $$\begin{array}{c} \begin{array}{cc} f_{1}\left(k_{1n},t\right)=e^{k_{1n}\;t} & f_{2}\left(k_{2n},t\right)=e^{k_{2n}\;t} \end{array}\text{.} \end{array}$$

Thus, when *t*=0, we have

(26) $$\begin{array}{c} f_{1}\left(k_{1n},0\right)=f_{2}\left(k_{2n},0\right)=1,\frac{\partial f_{1}\left(k_{1n},0\right)}{\partial t}=k_{1n},\frac{\partial f_{2}\left(k_{2n},0\right)}{\partial t}=k_{2n},n=0,1,2,3\ldots \text{ .} \end{array}$$

Then, equations (23) and (24) introduce the following systems:

(27) $$\begin{array}{c} \left[a_{0}+b_{0}\right]\cos\left(\omega_{0}x\right)+\overset{\infty }{\underset{n=1}{\sum }}\left[a_{n}+b_{n}\right]\cos\left(\omega_{n}x\right)=-\frac{q_{0}}{2LK\lambda^{2}}-\frac{\gamma Q_{met}}{2\lambda^{2}}-\frac{q_{0}}{KL}\overset{\infty }{\underset{n=1}{\sum }}\frac{1}{\left(\lambda^{2}+\omega_{n}^{2}\right)}\cos\left(\omega_{n}x\right)\text{,} \end{array}$$

and

(28) $$\begin{array}{c} \left[k_{10}a_{0}+k_{20}b_{0}\right]\cos\left(\omega_{0}x\right)+\overset{\infty }{\underset{n=1}{\sum }}\left[k_{1n}a_{n}+k_{2n}b_{n}\right]\cos\left(\omega_{n}x\right)=0\text{.} \end{array}$$

When *n*=0, we have

(29) $$\begin{array}{c} \omega_{0}=0,\cos\left(\omega_{0}x\right)=1,A_{10}=\frac{\varepsilon \tau_{q}+1}{\tau_{q}},A_{20}=\frac{\varepsilon }{\tau_{q}},{∆}_{0}={\left(\frac{\varepsilon \tau_{q}-1}{\tau_{q}}\right)}^{2}{,k}_{10}=\frac{-1}{\tau_{q}},k_{20}=-\varepsilon \text{.} \end{array}$$

Then, the system of equations (27) and (28) can be reduced to a system of equations as follows:

(30) $$\begin{array}{c} a_{0}+b_{0}+\overset{\infty }{\underset{n=1}{\sum }}\left[a_{n}+b_{n}\right]\cos\left(\omega_{n}x\right)=-\frac{q_{0}}{2LK\lambda^{2}}-\frac{\gamma Q_{met}}{2\lambda^{2}}-\frac{q_{0}}{LK}\overset{\infty }{\underset{n=1}{\sum }}\frac{1}{\left(\lambda^{2}+\omega_{n}^{2}\right)}\cos\left(\omega_{n}x\right)\text{,} \end{array}$$

and

(31) $$\begin{array}{c} -\frac{a_{0}}{\tau_{q}}-\varepsilon b_{0}+\overset{\infty }{\underset{n=1}{\sum }}\left[k_{1n}a_{n}+k_{2n}b_{n}\right]\cos\left(\omega_{n}x\right)=0\text{.} \end{array}$$

The equations (30) and (31) give two systems of algebraic equations as follows:

(32) $$\begin{array}{c} \begin{array}{c} \begin{array}{cc} a_{0}+b_{0}=-\left(\frac{q_{0}}{2LK\lambda^{2}}+\frac{\gamma Q_{met}}{2\lambda^{2}}\right), & a_{0}+\tau_{q}\varepsilon b_{0}=0, \end{array}\\ \begin{array}{ccc} a_{n}+b_{n}=\frac{-q_{0}}{LK\left(\lambda^{2}+\omega_{n}^{2}\right)}, & k_{1n}a_{n}+k_{2n}b_{n}=0, & n=1,2,3,\ldots \text{.} \end{array} \end{array} \end{array}$$

The solution of the abovementioned two systems gives the following equation:

(33) $$\begin{array}{c} \begin{array}{c} \begin{array}{cc} a_{0}=\left(\frac{\varepsilon \tau_{q}}{1-\varepsilon \tau_{q}}\right)\left(\frac{q_{0}}{2LK\lambda^{2}}+\frac{\gamma Q_{met}}{2\lambda^{2}}\right), & b_{0}=\left(\frac{-1}{1-\varepsilon \tau_{q}}\right)\left(\frac{q_{0}}{2LK\lambda^{2}}+\frac{\gamma Q_{met}}{2\lambda^{2}}\right), \end{array}\\ \begin{array}{ccc} a_{n}=\left(\frac{k_{2n}}{k_{1n}-k_{2n}}\right)\frac{q_{0}}{LK\left(\lambda^{2}+\omega_{n}^{2}\right)}, & b_{n}=\left(\frac{-k_{1n}}{k_{1n}-k_{2n}}\right)\frac{q_{0}}{LK\left(\lambda^{2}+\omega_{n}^{2}\right)}, & n=1,2,3\ldots . \end{array} \end{array} \end{array}$$

In this case, the exact solution of equation (6) takes the following form:

(34) $$\begin{array}{c} \theta \left(x,t\right)=\frac{q_{0}}{2LK\lambda^{2}}+\frac{\gamma Q_{met}}{2\lambda^{2}}+\frac{q_{0}}{KL}\overset{\infty }{\underset{n=1}{\sum }}\frac{1}{\left(\lambda^{2}+\omega_{n}^{2}\right)}\cos\left(\omega_{n}x\right)\\ +\left(\frac{1}{1-\varepsilon \tau_{q}}\right)\left(\frac{q_{0}}{2LK\lambda^{2}}+\frac{\gamma Q_{met}}{2\lambda^{2}}\right)\left[\varepsilon \tau_{q}e^{\frac{-t}{\tau_{q}}}-e^{-\varepsilon t}\right]\\ +\frac{q_{0}}{KL}\;\overset{\infty }{\underset{n=1}{\sum }}\left(\frac{k_{2n}e^{k_{1n}\;t}-k_{1n}e^{k_{2n}\;t}}{\left(k_{1n}-k_{2n}\right)\left(\lambda^{2}+\omega_{n}^{2}\right)}\right)\cos\left(\omega_{n}x\right)\text{.} \end{array}$$

For this case ∆<0, so, we have

(35) $$\begin{array}{c} f_{1}\left(k_{1n},t\right)=e^{\left(k_{1n}\;t\right)}\;\cos\left(\frac{\sqrt{-{∆}_{n}}}{2}\;t\right),f_{2}\left(k_{2n},t\right)=e^{\left(k_{2n}\;t\right)}\;\sin\left(\frac{\sqrt{-{∆}_{n}}}{2}\;t\right)\text{,}\\ \frac{\partial f_{1}\left(k_{1n},t\right)}{\partial t}=e^{\left(k_{1n}\;t\right)}\left[k_{1n}\cos\left(\frac{\sqrt{-{∆}_{n}}}{2}\;t\right)-\frac{\sqrt{-∆}}{2}\sin\left(\frac{\sqrt{-{∆}_{n}}}{2}\;t\right)\right]\text{,}\\ \frac{\partial f_{2}\left(k_{2n},t\right)}{\partial t}=e^{\left(k_{2n}\;t\right)}\left[k_{2n}\sin\left(\frac{\sqrt{-{∆}_{n}}}{2}\;t\right)+\frac{\sqrt{-{∆}_{n}}}{2}\cos\left(\frac{\sqrt{-{∆}_{n}}}{2}\;t\right)\right],\\ f_{1}\left(k_{1n},0\right)=1,f_{2}\left(k_{2n},0\right)=0,n=0,1,2,3\ldots ,\\ \frac{\partial f_{1}\left(k_{1n},0\right)}{\partial t}=k_{1n},\frac{\partial f_{2}\left(k_{2n},0\right)}{\partial t}=\frac{\sqrt{-{∆}_{n}}}{2},n=0,1,2,3\ldots ,\\ A_{10}=\frac{1}{\tau_{q}},A_{20}=\frac{\varepsilon }{\tau_{q}}{,∆}_{0}={\left(\frac{\varepsilon \tau_{q}-1}{\tau_{q}}\right)}^{2},k_{10}=\frac{-1}{\tau_{q}},k_{20}=-\varepsilon . \end{array}$$

By using the initial condition of equation (14), we get

(36) $$\begin{array}{c} a_{0}+\overset{\infty }{\underset{n=1}{\sum }}a_{n}\cos\left(\omega_{n}x\right)=-\frac{q_{0}}{2LK\lambda^{2}}-\frac{\gamma Q_{met}}{2\lambda^{2}}-\frac{q_{0}}{KL}\overset{\infty }{\underset{n=1}{\sum }}\frac{1}{\left(\lambda^{2}+\omega_{n}^{2}\right)}\cos\left(\omega_{n}x\right)\\ \left(-\frac{1}{\tau_{q}}\right)a_{0}+\left(\frac{\tau_{q}-1}{2\tau_{q}}\right)b_{0}+\overset{\infty }{\underset{n=1}{\sum }}\left[a_{n}k_{1n}+b_{n}\frac{\sqrt{-{∆}_{n}}}{2}\right]\cos\left(\omega_{n}x\right)=0\text{.} \end{array}$$

Solving the abovementioned system gives

(37) $$\begin{array}{c} a_{0}=-\left(\frac{q_{0}}{2LK\lambda^{2}}+\frac{\gamma Q_{met}}{2\lambda^{2}}\right),b_{0}=-\left(\frac{1}{\tau_{q}-1}\right)\left(\frac{q_{0}}{LK\lambda^{2}}+\frac{\gamma Q_{met}}{\lambda^{2}}\right)\text{,}\\ a_{n}=-\frac{q_{0}}{KL\left(\lambda^{2}+\omega_{n}^{2}\right)},b_{n}=\frac{2k_{1n}q_{o}}{KL\left(\lambda^{2}+\omega_{n}^{2}\right)\sqrt{-{∆}_{n}}}\text{.} \end{array}$$

Thus, in this case, the exact solution of equation (6) is [35]

(38) $$\begin{array}{c} \theta \left(x,t\right)=\frac{q_{0}}{2LK\lambda^{2}}+\frac{Q_{met}}{2}+\frac{q_{0}}{KL}\overset{\infty }{\underset{n=1}{\sum }}\frac{1}{\left(\lambda^{2}+\omega_{n}^{2}\right)}\cos\left(\omega_{n}x\right)-\left(\frac{q_{0}}{2LK\lambda^{2}}+\frac{\gamma Q_{met}}{2\lambda^{2}}\right)e^{-t/\tau_{q}}\;\cos\left(\frac{\sqrt{-{∆}_{0}}}{2}\;t\right)\\ -\left(\frac{1}{\tau_{q}-1}\right)\left(\frac{q_{0}}{LK\lambda^{2}}+\frac{\gamma Q_{met}}{\lambda^{2}}\right)e^{-t}\;\sin\left(\frac{\sqrt{-{∆}_{0}}}{2}\;t\right)\\ +\frac{q_{0}}{KL}\;\overset{\infty }{\underset{n=1}{\sum }}\left[e^{\left(k_{1n}\;t\right)}\;\cos\left(\frac{\sqrt{-{∆}_{n}}}{2}\;t\right)+\frac{2k_{1n}}{\sqrt{-{∆}_{n}}}e^{\left(k_{2n}\;t\right)}\;\sin\left(\frac{\sqrt{-{∆}_{n}}}{2}\;t\right)\right]\frac{\cos\left(\omega_{n}x\right)}{\left(\lambda^{2}+\omega_{n}^{2}\right)}\text{.} \end{array}$$

Now, we will consider that the skin tissue has been considered to be subjected to a thermal shock for a small value of time *t*_0_ due to the thermal effect of an electrical shock with a constant voltage V (V) and a constant resistance of the skin tissue *R*_*e*_(Ω) [36].

According to Joule's equation of electrical heating, we have [36]

(39) $$\begin{array}{c} q_{0}=\frac{V^{2}t_{0}}{R_{e}}\text{,} \end{array}$$

where *t*_0_(*t*_0_ > 0) is the time interval parameter of the electrical shock.

## 4. Results and Discussions

The temperature distribution of skin tissue is examined by using three bioheat transfer models (V-C, Pennes, and Tzou).  Table 1 shows the values of the pertinent thermal parameters that were used in the numerical results and calculations for this project [17, 23, 24, 30–32, 35, 39].

**Figure 2:**
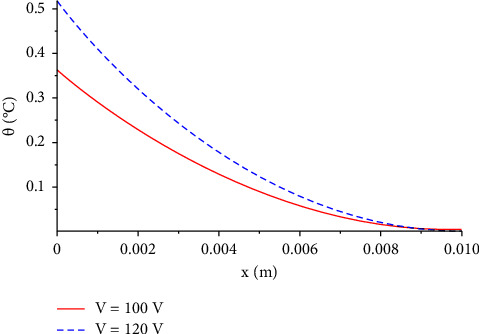
The function of temperature increment with different values of voltage when *R*_*e*_=300 Ω, *t*=300 s, *t*_0_=1.0 s, *τ*_*T*_=20 s, and *τ*_*q*_=20 s.

**Figure 3:**
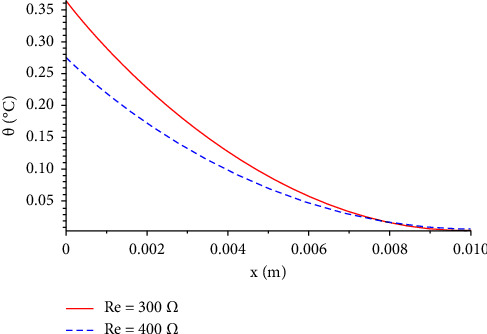
The function of temperature increment with different values of resistance when *V*=100 V, *t*=300 s, *t*_0_=1.0 s, *τ*_*T*_=20 s,  and *τ*_*q*_=20 s.

**Figure 4:**
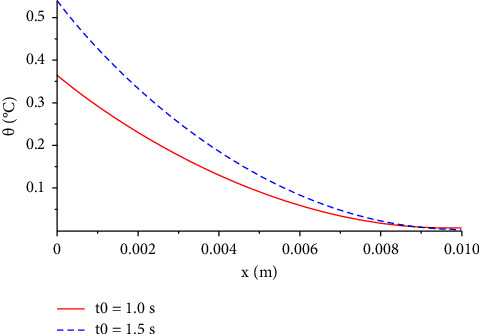
The function of temperature increment with different values of electric shock time when *V*=100 V, *R*_2_=300 Ω, *t*=300 s, *τ*_*T*_=20 s, and *τ*_*q*_=20 s.

**Figure 5:**
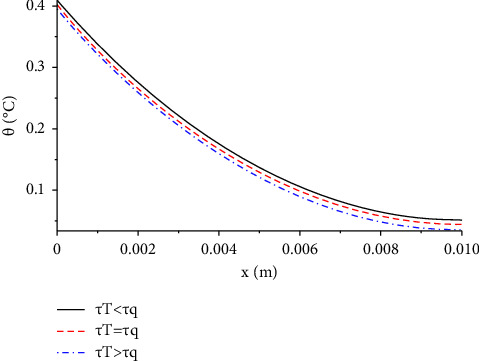
The function of temperature increment with different values of lag-time parameters when *V*=100 V, *R*_2_=300 Ω, *t*=300 s, and *t*_0_=1.0 s.

**Figure 6:**
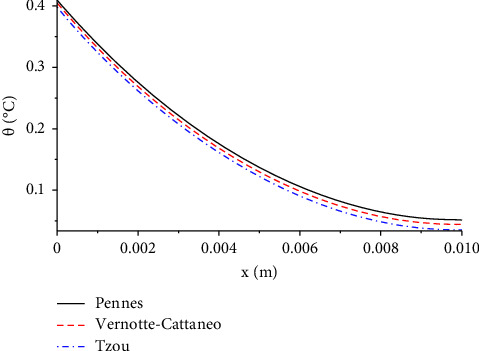
The function of temperature increment with the three bioheat models when *V*=100 V, *R*_2_=300 Ω, *t*=300 s, and *t*_0_=1.0 s.

In Figures 2–6, we plot the solutions of the heat conduction temperature given in equation (38) versus the length. All figures from 26 seem to have the same behaviour, which is that the temperature decreases rapidly as the length increases. Specifically, in Figure 2, we compare the temperature increment distribution for the voltage values V=100 V, and V=120V. It is clear from the figure that as the value of the voltage increases, the temperature will rise when the length is less than 0.008. However, there is no big difference shown in temperature after this point.

We also studied the influence of resistance when *R*_*e*_=300 Ω and *R*_*e*_=400 Ω in the temperature increment distribution, as shown in Figure 3. It clearly shows that the high resistance plays a significant role in reducing the temperature when the length is less than 0.008. This result seems to agree with what is expected intuitively.

Figure 4 shows the temperature increment distribution for the two different values of electric shock time, *t*_0_=1.0 s, and *t*_0_=1.5 s. We noted that as the electric shock time increases, the temperature increment distribution will also rise which intuitively makes sense.

We can compare this figure with Figure 8 in [26], where both figures represent the same result and the same attitude of the temperature increment based on the different values of the ramp-time heat parameter.

In Figure 5, we compare the temperature increment distribution considering the three cases of relaxation time parameters, *τ*_*T*_, *τ*_*q*_. When *τ*_*T*_ < *τ*_*q*_, the temperature declines slightly and uniformly over the *x*-axis whereas the temperature will start to increase somewhat if the value of *τ*_*T*_ exceeds or is equal to the value of *τ*_*q*_. The values of the temperature increment take the following order:

(40) $$\begin{array}{c} \theta \left(\tau_{T}>\tau_{q}\right)>\theta \left(\tau_{T}=\tau_{q}\right)>\theta \left(\tau_{T}<\tau_{q}\right)\text{.} \end{array}$$

Figure 6 compares the distribution of the temperature change for three different bioheat models, which are Pennes, Vernotte–Cattaneo, and Tzou. The Tzou model plays a considerable role in reducing the temperature. The values of the temperature increment based on the three studied models take the following order:

(41) $$\begin{array}{c} \theta \left(\text{Pennes}\right)>\theta \left(\text{Vernotte}-\text{Cattaneo}\right)>\theta \left(Tzou\right)\text{.} \end{array}$$

Equation (41) shows that the profile of the speed of the thermal wave through the skin tissue is decreasing from Pennes's model, Vernotte–Cattaneo, to Tzou. In other words, Tzuo's model ensures that the thermal wave transfer through the skin tissues at finite or limited speeds is better than other models, thus making this model the best and closest to the reality of the physical behaviour of the skin than other models. So, depending on the lag-time parameters of temperature gradient and heat flux makes the model of Tzou more successful and more useful than the other models.

**Figure 7:**
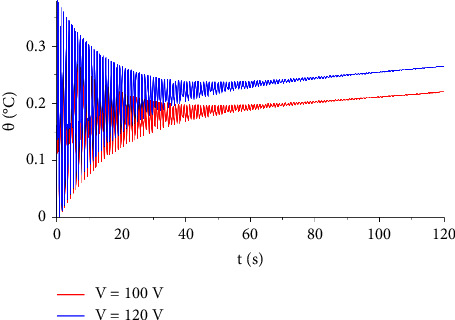
The function of temperature increment with different values of voltage when *R*_*e*_=300 Ω, *x*=0.001 m, *t*_0_=1.0 s, *τ*_*T*_=20 s, and*τ*_*q*_=20 s.

**Figure 8:**
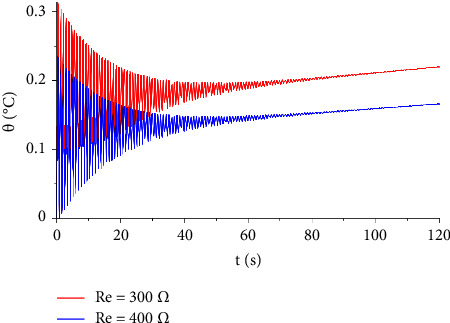
The function of temperature increment with different values of resistance when *V*=100 V, *x*=0.001 m, *t*_0_=1.0 s, *τ*_*T*_=20 s, and *τ*_*q*_=20 s.

**Figure 9:**
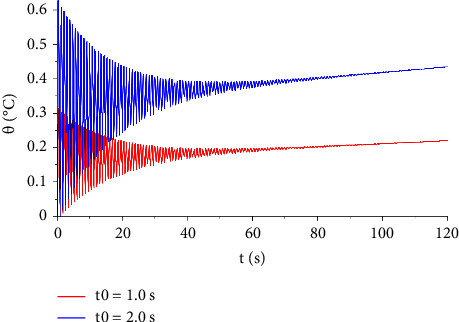
The function of temperature increment with different values of electric shock time when *V*=100 V, *R*_2_=300 Ω, *x*=0.001 m, *τ*_*T*_=20 s, and *τ*_*q*_=20 s.

**Figure 10:**
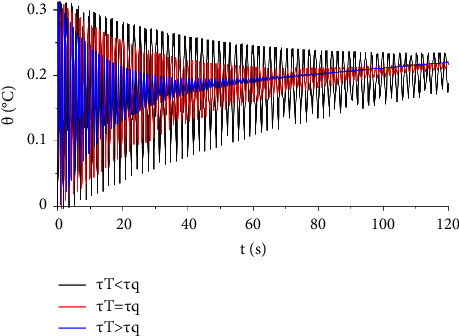
The function of temperature increment with different values of lag-time parameters when *V*=100 V, *R*_2_=300 Ω, *x*=0.001 m, and *t*_0_=1.0 s.

**Figure 11:**
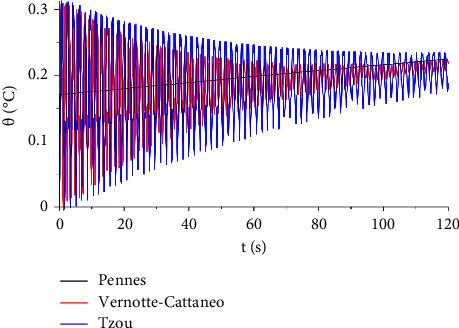
The function of temperature increment with the three bioheat models when *V*=100 V, *R*_2_=300 Ω, *x*=0.001 m, and *t*_0_=1.0 s.

Then, we studied the behaviour of temperature increment distribution over time to understand as to what extent can other factors such as voltage, resistance, electric shock time, and relaxation parameters influence the temperature, and this is shown in Figures 7–11.

Figures 7–11 show the deep fluctuation in temperature at a high altitude in the first few seconds. Then, it gradually stabilizes into a periodic motion as time increases.

In Figure 7, the distribution of the temperature increment is illustrated for two values of voltages, V=100, and V=120. It indicates that the voltage has a significant influence on the temperature increment distribution. Moreover, increasing resistance and shock time seem to have a non-neglected effect on the temperature increment distribution either by increasing or decreasing the temperature, respectively, and this is explained in Figures 8 and 9.

To study the influences of time relaxation parameters on the temperature, we plotted the temperature increment distribution over time considering three cases: *τ*_*T*_ < *τ*_*q*_, *τ*_*T*_=*τ*_*q*_, *τ*_*T*_ > *τ*_*q*_ . It is clear from Figure 10 that the temperature fluctuates from the highest to lowest temperature over time when *τ*_*T*_ < *τ*_*q*_ at a high altitude whereas the temperature zigzags fast at a high altitude at the beginning and then declines significantly over time as *τ*_*T*_ ≥ *τ*_*q*_. Moreover, the attitude of the temperature increment in Figure 10 is the same as in Figures 3 and 7 in Ezzat [26].

Figure 11 compares the behaviour of temperature increment distribution over time between three different models: Pennes, Vernotte−Cattaneo, and Tzou. Among these models, Tzou seems to illustrate the lowest temperature increment with the smallest altitude.

**Figure 12:**
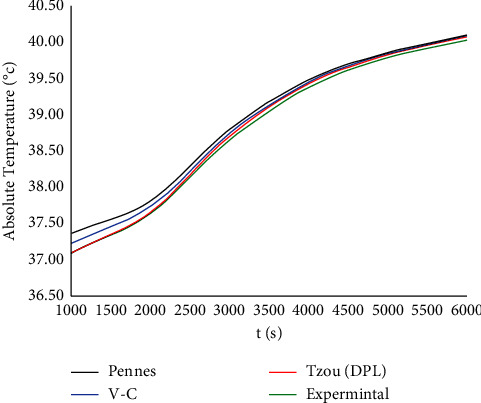
The function of temperature increment with the three bioheat models when *V*=100 V, *R*_2_=300 Ω, *x*=0.025 m, and *t*_0_=10.0 s.

For validation and to know which model is more close to the real behaviour of the skin tissue, Figure 12 has been performed with conditions similar to the experimental condition in [40] to compare the behaviour of temperature increment distribution over a long interval of time between the three different models: Pennes, Vernotte−Cattaneo, Tzou, and the experimental and cited results in [40]. Thus, this figure shows that Tzou is the best model where the temperature increment based on this model is closer to the experimental results more than the other models.

## 5. Conclusion

The temperature interaction and the response of the skin tissue caused by a continuous flow of surface heat caused by a constant voltage electrical current was the goal of this study. The bioheat transfer of the dual-phase-lag (DPL) model has a precise analytical solution. It is used to separate the variables into a finite domain for the governing equations. The reactions at the transition temperature have been measured and studied. Figures have been provided that compare the Pennes, Vernotte−Cattaneo, and Tzou models.

The results concluded the following:

- When the value of the voltage increases, the temperature rises.
- When the value of the resistance decreases, the temperature rises.
- When the electric shock time increases, the temperature increment distribution also rises.
- When the value of the temperature relaxation time increases *τ*_*T*_ more than the value of the gradient temperature relaxation time *τ*_*q*_, the temperature increment distribution rises.
- The temperature increment based on Penne's model is greater than its values based on the Vernotte–Cattaneo model, Tzou's model.
- The current results show that the thermal waves based on Tzou spread at a finite speed in the skin tissue which removes the Vernotte–Cattaneo and Pennes's shortcomings.
- Depending on the lag-time parameters of temperature gradient and heat flux makes the model of Tzou more successful and more useful than the other models.

## Figures and Tables

**Table 1:** The skin and blood materials properties.

Parameters	Units	Skin
*K*	W/m*·*°C	0.215
*ρ*	kg/m^3^	1,000
*ρ*_*b*_	kg/m^3^	1,060
*C*	J/kg*·*°C	4,187
*C*_*b*_	J/kg*·*°C	3,800
*W*_*b*_	ml/C m	0.00052
*T*_*b*_	°C	37
*τ*_*T*_	s	10
*τ*_*q*_	s	30
*L*	m	0.01

## Data Availability

No data were used to support the study.
